# Evaluation of the results of patients who applied to the Çukurova University, Medical Genetics Department for prenatal diagnosis and determination of genetic counseling principles

**DOI:** 10.3906/sag-2004-298

**Published:** 2020-04-30

**Authors:** Sevcan TUĞ BOZDOĞAN, Selim BÜYUKKURT, Sinem ÖZER, Atıl BİŞGİN

**Affiliations:** 1 1Department of Medical Genetics, Faculty of Medicine, Çukurova University, Adana Turkey; 2 Adana Genetic Disease Diagnosis and Treatment Center (AGENTEM), Çukurova University Adana Turkey; 3 Department of Obstetrics and Gynecology, Faculty of Medicine, Çukurova University, Adana Turkey

**Keywords:** Prenatal diagnosis, cytogenetics, molecular testing, molecular cytogenetics, genetic counseling

## Abstract

**Background/aim:**

The aim of this study was to summarize the experiences of a single medical center for genetic diagnosis and treatment of prenatal patients.

**Materials and methods:**

This study includes a retrospective data analysis of 2843 prenatally investigated cases using invasive methods during a 6-year period (2013–2019) at a single tertiary care center.

**Results:**

Chromosomal abnormalities were detected in 80 out of 1221 amniotic fluid samples;,178 out of 1608 chorionic villus samples, and 1 out of 14 cordocentesis samples. The most common chromosomal abnormality was trisomy 21. At least one mutation was detected in 63 of the 152 molecular tests performed on fetuses.

**Conclusion:**

Clinical procedures such as ultrasounds and genetic tests are able to provide a better clinical follow-up for pregnant women about the possible congenital anomalies or any genetic condition, with proper genetic counseling and testing methodology.

## 1. Introduction 

Prenatal diagnosis has become widely available in the last decade and an increasing variety of anomalies can be detected via both chromosomal analysis and DNA-based molecular methods. There are two broad types of tests for genetic disorders: screening tests, which evaluate the risk of a fetus for certain genetic disorders and diagnostic tests, which can detect specific genetic disorders actually present in the fetus. Thus, the practice of prenatal screening is an important step for the identification of changes, resulting in the rapid expansion in genomic testing.

There is a plethora of genetic disorders that can be diagnosed during pregnancy. These disorders are classified as chromosomal anomalies, monogenic diseases, complex disorders, and teratogenic disorders. Monogenic disorders occur as a result of mutation(s) in a single gene. Although the majority of genetic diseases are caused by single gene mutations, the numerical and structural chromosomal abnormalities are the most evaluated causes of fetal anomalies and congenital genetic disorders [1]. The early and precise diagnosis of such anomalies during pregnancy is the main goal of prenatal diagnostics. The most common aneuploidies observed in prenatal diagnosis during the second trimester are the trisomies of chromosomes 13, 18, or 21, and the gonosomal abnormalities. Trisomy 13, 18, and 21 account for 89% of autosomal chromosomal aneuploidy pregnancies, which can survive to term. However, these patients mostly present with severe phenotypes [2]. Aneuploidies are traditionally detected by full karyotype analysis of cultured cells collected, which has a very high diagnostic accuracy up to 99.8%. Karyotype analyses are, therefore, the reference method for invasive prenatal diagnosis of fetal chromosomal abnormalities. Rapid detection of these aneuploidies following sampling via amniocentesis is also achievable with fluorescence in situ hybridization (FISH) by the utilization of centromere or locus-specific probes. All these assays can be performed via amniotic fluid or chorionic villus sampling (CVS) by an experienced specialized clinician.

## 2. Materials and methods

### 2.1. Patients 

A retrospective survey covering the 6-year period from 2013 to 2019 at the Medical Genetics Department and Adana Genetic Diseases Diagnosis and Treatment Center (AGENTEM) of **Ç** ukurova University includes prenatal analysis of CVS, amniocentesis, and cordocentesis in 2843 pregnancy cases. 

The cytogenetic and molecular data of the 2843 pregnancies were obtained from amniocentesis (AS) (1221), CVS (1608), and cordosentesis (CS) (14). Prenatal cases in which abnormalities were detected were referred to the medical genetics department. Conventional prenatal karyotypic analyses were performed for all of the subjects. All of the patients underwent an invasive prenatal diagnosis both received pre- and posttest counseling from an experienced genetic counselor and/or medical geneticist.

The study protocol was approved by the local ethics committee of the study center with a protocol number of 13/68 (Çukurova University, Medical Faculty, Noninterventional Clinical Research Ethics Committee, 2017).

### 2.2. Methods

The main indications for chromosome analysis were advanced maternal age, increased risk based on maternal serum screening tests, and abnormal ultrasound findings of chromosomal aberrations. Moreover, an affected child or a pedigree analysis revealing a positive familial history are the indicators of molecular testing.

Conventional karyotype analysis was performed on cultured biological samples following the European Cytogeneticists Association Guidelines [3]. The chromosomal abnormality determination was performed in two simultaneously cultured flasks, which were made for each patient sample, and at least 20 metaphases were analyzed at the level of 450–550 banding. The FISH analysis was carried out with commercially available probes according to the manufacturers’ protocols. Specific molecular analyses were performed on DNA isolated from samples, based on family history and predefined mutations. 

## 3. Results

A total of 2843 fetal materials were examined and chromosomal analyses were performed in all cases. Fetal material cell culture failure occurred in only 3 cases; these cases were excluded from the statistical data. Chromosomal abnormalities were detected in 80 of 1221 amniotic fluid samples, 178 of 1608 CVS samples, and in 1 of 14 cordocentesis. The most frequent chromosomal abnormality was trisomy 21 (Down syndrome) from both CVS and amniocentesis samples (Table 1). 

**Table 1:** The distribution of abnormal karyotype results.

Sample type	Sample number	The number with a normal karyotype	The number with an abnormal karyotype	Karyotype (N)
AS	1221	1141	80	47,--,+21 (N = 32) (40% of AS with chromosomal anomalies)
				47,--,+18 (N = 15)
				47,--,+13 (N = 4)
				69,--- (N = 7)
				Other N = 22
CVS	1608	1430	178	47,--,+21 (N = 68) (38.2% of CVS with chromosomal anomalies)
				47,--,+18 (N = 39)
				45,X (N = 4)
				47,--,+13 (N = 14)
				69,--- (N = 3)
				Other N = 34
CS	13	12	1	47,XX,+18

The FISH technique was used to evaluate the microdeletion syndromes via syndrome-specific region probes in 28 pregnancies while 26 of 28 were examined for Di George syndrome, only 1 was analyzed for Prader–Willi syndrome and another 1 for Cri-du-Chat syndrome. A deletion was detected in the Cri-du-Chat FISH test, but the rest were reported as normal. 

FISH was also used to characterize 131 samples for rapid aneuploidy, of which 94 were determined to be normal. Of the 37 samples with a FISH-identified chromosomal abnormality, 20 were from AS specimens and 17 were from CVS specimens (Table 2). 

**Table 2:** Rapid aneuploidy FISH results with incidences.

Material type	Total analysis with abnormal result	Mutation detection rate	FISH result (N)
AS	20	20/131	Trisomy 21 (N = 10)
			Trisomy 18 (N = 9)
			Triploidy (N = 1)
CVS	17	17/131	Trisomy 21 (N = 10)
			Trisomy 18 (N = 5)
			Trisomy 13 (N = 1)
			Turner S (N = 1)

Molecular testing was performed in 152 cases which revealed 41.45% positivity rate (n = 63). The numbers together with the most frequent genes and the mutations detected are listed in Table 3 (all the mutations in related genes are listed in the supplemental data (Table S1)). 

**Table 3:** The distribution of materials tested for specific genes and the list of the most frequent mutations detected.

Material type	Total analysis	Mutation detected	Gene	Total number of tested samples	Total number of samples with mutation	Total number of samples with no mutation
AS/ CVS/ CS	152	63	SMA	17	4 homozygous	13
			CFTR	12	2 heterozygous1 homozygous	9
			PAH	9	5 heterozygous1 homozygous	3

## 4. Discussion 

Genetic counseling is defined as a communication process with individuals and their families about the genetic diseases with the aim of risk reducing for the recurrence, mostly through the provision of both prenatal and postnatal diagnosis options and offering the most current therapies together with psychotherapeutic support [4]. Nondirectiveness is the main and basic principle that requires the maintenance of a neutral stance of genetic counselor in order to support and also to respect the patient’s personal values and decisions [4,5]. 

A prenatal diagnosis can be either invasive or noninvasive. Invasive prenatal sampling is necessary to apply molecular methods if the results of genetic testing has an impact on clinical decision-making and clinical outcomes such as when the mother is a carrier for X-linked diseases, or if both parents are carriers for an autosomal recessive disorder, as well as when either of the parents is afflicted with an autosomal dominant disease. 

It is important to not forget that the prenatal genetic testing is an optional strategy that depends on the patient’s needs and decisions. The patients should be informed about what the tests can reveal, before the sampling procedures, as well as after the results are in. The major supporting tool for a medical geneticist is the careful pre- and posttest counseling [6].

According to the literature, Down syndrome (trisomy 21) is the most common chromosomal abnormality, with an incidence estimated one in 700–1000 live births. It is considered to be one of the major congenital causes of intellectual disability in the human population. In our study group, numerical chromosomal anomalies were detected in 80 of the AS-diagnosed patients and Down syndrome accounted for 32 (40%) of them. Chromosomal anomalies were found in 178 of the CVS-sampled patients, and Down syndrome was diagnosed in 68 (38.2%) of them. In previous studies, the rate of chromosomal abnormalities had been found lower than that in our study, but reported series were also in accordance with our results as the most common anomaly was the Down syndrome [7,8]. The higher rates in our study depend on both the sensitivity of screening tests applied to all pregnant women to predict Down syndrome and a well-established clinical algorithm of perinatology and genetic counseling together with clinical follow-ups. The other main reason is the fact that our center and the hospital give a healthcare service as a reference center for prenatal diagnosis and a main hub for genetic counseling, from children to adults. As prenatal screening and diagnostic techniques have become more enhanced and widely available, medical geneticists should expect to provide information and support following a new diagnosis of Down syndrome on a frequent basis. If the fetus is diagnosed with Down syndrome during the prenatal period, the common problems in Down syndrome should be explained to the family clearly, but the decision should be made by the family as to whether to terminate the pregnancy.

It has been estimated that a minimum 0.05% of newborns have unbalanced structural chromosomal abnormalities, which encompasses pathological alterations resulting from the breakage or exchange of chromosome material [9,10]. However, there were no fetuses with structural chromosomal anomalies detected among our case series. 

All of the chromosomal aberrations we detected were previously known alterations; therefore, the clinical outcomes were predictable and the content of genetic counseling was clear. Uncommon chromosomal abnormalities in prenatal diagnosis require correlations between the cytogenetic aberrations and ultrasonography findings. These rare chromosomal abnormalities are very important for providing easy to understand genetic counseling for the parents [6]. 

Prior to molecular prenatal testing, genetic counseling should be offered to discuss the information ratio and limitations of prenatal testing along with the consequences of both test results with or without abnormalities (mutations). Hence, a normal prenatal test result cannot absolutely exclude the diagnosis of a genetic disease. 

Molecular tests were performed as prenatal tests in a 152-person pregnant group. Sixty three of them identified a mutation in the fetus. The most frequent tested genes were spinal muscular atrophy (SMA), cystic fibrosis (CFTR), and phenylketonuria (PAH). All of the families with specific molecular testing had a predetermined history of disease and mutations in the parents had been identified before prenatal testing. The genetic counseling and the family’s understanding of the disease was easier due to familial history in this group.

The advances in prenatal diagnosis enabled us to plan the antenatal care and newborn units, as well as the delivery. 

Together, our data indicates the general acceptance, carrier frequencies, and prenatal testing results in the south-east Mediterranean region of the Turkish population. Moreover, our study describes the first comprehensive frequencies and risks from a large cohort tested from a cytogenetic and molecular genetic perspective in the region. This data can serve as a reference for future screening and genetic counseling. 

The acceptance of prenatal screening by the efforts of medical geneticists also offers insights into the future potential for whole exome or genome sequencing of all known mutations causing serious diseases of newborns. Thus, preventive medicine is also one of the main aims for all health systems, which includes simultaneous screening of both partners, in conjunction with prescreening genetic counseling. 

Supplementary Materials
