# Supplementary material for: Evaluation of the results of patients who applied to the Çukurova University, Medical Genetics Department for prenatal diagnosis and determination of genetic counseling principles
Source: Turk J Med Sci. 2020 Apr 30;51(2):657–60. doi: 10.3906/sag-2004-298 (PMC8203124; doi:10.3906/sag-2004-298)
Supplement: Supplementary file 1 — Supplementary Materials [file turkjmedsci-51-657-sup001.pdf]

Table S1. The full list of the genes and mutations detected by prenatal tests.

Gene	Mutation	Sample (number of the mutation)
SMA1	Exon 7–8 deletion (4 homozygous)	CVS (4)
PAH	c.1243G>A p.D415N (het)	CVS (4) AS (2)
	c.165delT p.F55Lfs*6 (het)	
	c.1066–11G>A IVS10-11G>A (het)	
	c.1199+1G>C IVS11+1G>C (het)	
	c.782G>A p.R261Q (het)	
	Exon 3 DEL (homozygous)	
PCCA	c.2010-2A>G IVS22-2A>G(het) (hom)	CVS (4)
	c.1746G>A p.S582S (2 hom)	
BETA G.	c.20A>T p.E6V / c.-101C>T (2 comp het)	AS (3) CVS (1)
	IVS-I110 G>A / Hb S A-T (comp het)	
	c.93-21G>A IVS1-110G>A (hom)	
CFTR	c.2657+5G>A IVS14b+5G>A (het)	CVS (1) AS (2)
	c.3659C>T p.T1220I (het)	
	c.1521_1523delCTT p.508delF (hom)	
GALC	c.489_490delGC p.W163Cfs*24 (het)	CVS (3)
	c.1807G>T p.G603* (het) (hom)	
ARSB	c.962T>C p.L321P (2 hom)	CVS (3)
	c.1036delG p.E346Sfs* (het)	
HEXB	c.149_158del CCAAGCCGGG (het)	CVS (2)
	c.1083-2A>G IVS8-2A>G (hom)	
MUT	c.668A>G p.K223R (hom)	CVS (1) AS (1)
	c.1843C>A p.P615T (het)	
GCDH	c.743C>T p.P248L (het)	CVS (2)
	c.1228G>A p.V410M (het)	
GAA	c.2237G>A p.W746* (hom)	CVS (2)
	c.1195-17_1199del pD399Pfs*105 (het)	
ASAH1	c.92G> p.C37F (het) (hom)	CVS (1) AS(1)
PRF1	c.1122G>A p.W374* (het) (hom)	CVS (2)
CTNS	c.451A>G p.R151G (2 het)	AS (2)

Table S1. (continued)

Gene	Mutation	Sample (number of the mutation)
ABCD4	c.1093G>T p.G365C / c.1411C>T p.R471W (het)	AS
AGPAT2	c.514G>A p.E172K (het)	KS
ALMS1	c.6828C>G p.C2276* (het)	AS
ANTXR2	c.945T>G p.C315W (het)	CVS
ATM	c.6047A>G p.D2016G (het)	CVS
BBS7	c.947G>T p.G316V (hom)	CVS
CRTAP	c.535G>T p.E179* (het)	CVS
DMD	45-49 DEL (hom)	AS
EIF2B3	c.833A>G p.Y278C (het)	AS
ETHE1	c.487C>T p.R163W (het)	AS
FAH	c.554-1G>T IVS6-1G>T (het)	CVS
GALT	c.1046T>G p.L349R (het)	AS
GLB1	c.8G>T p.G3V (hom)	AS
GNPTAB	c.232_234delGTT p.77delV (het)	CVS
IDS	c.253G>A p.A85T (het)	CVS
IVD	c.157C>G p.R53G (het)	AS
NAGLU	c.733T>C p.F245L (het)	CVS
PHKA2	c.759A>C p.K253N (hem)	CVS
TBC1D20	c.665_669TCACC p.I222Mfs*8 (het)	CVS
HBA1-HBA2	(het) (hom)	CVS (2)

Het: heterozygous, hom: homozygous.
